# The Auspicious Opening of the First Regular Session of the Dental Department of the University of Maryland

**Published:** 1882-10

**Authors:** 


### EDITORIAL, ETC.

*The Auspicious Opening of the first Regular Session of the Dental Department of the University of Maryland* on the 2nd day of October 1882, is not only extremely gratifying to all the friends of this new institution, but is unprecedented.

Beginning the lectures, clinics and demonstrations on the first or opening day of the session with *forty students present and matriculated*, the number steadily increased until at the middle of the month of October there were nearly *sixty* matriculants enrolled as dental students in the Dental Department of the University of Maryland, with quite a number yet to come by the 1st of November following. And what is still more gratifying than the mere number of matriculants is the fact that the class of students in attendance is *a very superior one.* A number of them have attended lectures etc., during former sessions in several other dental institutions, while others have been in practice for five eight and ten years; and all, without a single exception, express their satisfaction and pleasure concerning the course of study, clinical advantanges and the many facilities presented by this Dental Department for complete instruction in all the branches of Dental Science.

Another very gratifying fact to both the faculty and students is the rapid growth of the Infirmary and Laboratory practice. Already this practice, on account of the numerous sources at command, has reached such proportions, that the large number of new Operating Chairs are daily filled with patients upon whom the students operate, thereby affording them the actual experience and skill so necessary to the future successful practice of dentistry.

The arrangements and equippments of the new Dental Infirmary and Laboratory Building are so complete as to command the highest commendations from the many visitors. Indeed everything connected with the Dental Department of the University of Maryland is working in the most harmonious and successful manner.

